# Optimization of Wall Material of Freeze-Dried High-Bioactive Microcapsules with Yellow Onion Rejects Using Simplex Centroid Mixture Design Approach Based on Whey Protein Isolate, Pectin, and Sodium Caseinate as Incorporated Variables

**DOI:** 10.3390/molecules27238509

**Published:** 2022-12-03

**Authors:** Elham Azarpazhooh, Parvin Sharayei, Xin Rui, Mehranoosh Gharibi-Tehrani, Hosahalli S. Ramaswamy

**Affiliations:** 1 Agricultural Engineering Research Department, Khorasan Razavi Agricultural and Natural Resources Research and Education Center, AREEO, Mashhad P.O. Box 91735-488, Iran; 2 College of Food Science and Technology, Nanjing Agricultural University, 14 1 Weigang Road, Nanjing 211306, China; 3 Department of Food Science & Technology, Sabzevar Branch, Islamic Azad University, Sabzevar 9618956878, Iran; 4 Department of Food Science and Agricultural Chemistry, Macdonald Campus of McGill University, 21111 Lakeshore Road, Ste. Anne de Bellevue, QC H9X 3V9, Canada

**Keywords:** encapsulation, onion rejects/waste, optimization, bioactives, simplex-lattice mixture design

## Abstract

For the food sector, onion rejects are an appealing source of value-added byproducts. Bioactive compounds were recovered from yellow onion rejects using a pulse electric field process at 6000 v and 60 pulses. The onion extract was encapsulated with whey protein isolate (WPI), pectin (P), and sodium caseinate (SC) with a mass ratio of 1:5 (extract/wall material, *w*/*w*). A Simplex lattice with augmented axial points in the mixture design was applied for the optimization of wall material for the encapsulation of onion reject extract by freeze-drying (FD). The optimal wall materials were 47.6 g/100 g (SC), 10.0 g/100 g (P), and 42.4 g/100 g (WPI), with encapsulation yield (EY) of 85.1%, total phenolic content (TPC) of 48.7 mg gallic acid equivalent/g DW, total flavonoid content (TFC) of 92.0 mg quercetin equivalent/g DW, and DPPH capacity of 76.1%, respectively. The morphological properties of the optimal encapsulate demonstrated spherical particles with a rough surface. At optimal conditions, the minimum inhibitory concentration (MIC) of the extract (mean diameter of inhibition zone: 18.8 mm) was shown as antifungal activity against *Aspergillus niger*.

## 1. Introduction

Natural antioxidants that can replace synthetic ones used in food products have gotten a lot of attention in recent years [1]. Anti-inflammatory, anti-allergenic, antibacterial, antioxidant, and other physiological effects have been discovered with incorporated phenolic compounds present in various plants [2].

Particularly, the onion is abundant in quercetin, quercetin glucosides, quercetin polymers, ferulic acid, gallic acid, and kaempferol, which have significant health benefits associated with contributing to biological activities like anti-diabetic, antioxidant, anti-inflammatory, anti-cancer, anti-microbial, and enzyme inhibitory effects [3]. Some traditional methods are commonly utilized to recover essential components from plant matrices; however, new concepts are being explored as the food industry emphasizes the need for ecologically friendly and sustainable operations. Onions, onion byproducts, and onion rejects have been used as natural antioxidant food ingredients. An innovative method for producing natural and useful components is to maximize the recovery of phytochemicals from onion rejects [4].

The pulsed electric field is a demonstrated approach that includes applying high-voltage electrical pulses to the food product between two electrodes (typically 20–80 kW). The use of a high-intensity pulsed electric field in the extraction of important chemicals has been shown to be safe [5]. Because the extract is high in antioxidant and antibacterial compounds, and their stability in various environmental circumstances is limited, it is vital to choose a method that can protect these compounds from environmental influences while also allowing for their timely and complete release. Microencapsulation is one approach to safeguarding active and sensitive components during the extraction process. A procedure in which a substance or mixture of chemicals is coated or enclosed by other substances is known as microencapsulation. Microencapsulation of effective constituents is widely used in the food industry for a variety of reasons, including ease of transportation, increased shelf life of the final product, reduced evaporation, decomposition, or reaction with other food compounds during the process, improved food safety, and controlled release of effective constituents. Choosing the right wall material and drying procedure is critical for the success of the microencapsulation process [6]. In the encapsulation process, the wall material is extremely important. Polysaccharides (starches, maltodextrins, and gum Arabic), lipids (stearic acid, mono- and diglycerides), and proteins (gelatin, casein, milk serum, soy, and wheat) are among the various types of wall materials that have been widely explored, and the structure and properties of each coating agent impart different physicochemical properties to the encapsulate [7].

Proteins are efficient carriers for biochemicals due to their low cost, biodegradability, and functionalities that allow them to form films, gels, emulsifiers, and hold onto water [8]. Whey protein (WP) has high nutritional qualities (e.g., great protein content with abundant essential amino acids) and excellent bioavailability. Additionally, β-lactoglobulin and α-lactalbumin (the main components of whey protein) provide good emulsification and protective film-forming abilities that meet the demands of encapsulation [9]. Whey protein isolate (WPI) has hydrophobic and hydrophilic amino acids that facilitate the encapsulation of hydrophobic compounds [10].

Fundamental research has been conducted on the encapsulation characteristics of sodium caseinate in spray-dried emulsions, including functional lipophilic food components [11]. Sodium caseinate is a water-soluble milk protein with an excellent amphiphilic property, meaning it includes both hydrophobic and hydrophilic groups, making it ideal for both emulsification and encapsulation [12]. The highly disordered structure of sodium caseinate makes it easy to rapidly adsorb onto the oil/water interface and effectively lower the interfacial tension, thus stabilizing the oil droplets in the aqueous phase via electrostatic stabilization [13]. Sodium caseinate contains a large number of hydrophilic and hydrophobic groups that attract each other with water and fat equivalents, respectively, giving it emulsifying and stabilizing properties [14]. Another extensively utilized wall material is pectin, which is a structural heteropolysaccharide. Pectin (P) is made up of D-galacturonic acid units linked together by α-(1–4) glycosidic bond [15]. The carboxylic groups of these uronic acids may be esterified by methyl groups. At some distinguishing locations, galacturonic acid is substituted by -(1–2)-linked L-rhamnose units [16]. The molecular weight and degree of esterification of pectin determine its suitability for use as a wall material in encapsulation techniques, drug delivery systems, and controlled release formulations [17]. Many studies have been published on the usage of low-methoxyl (LMP) and high-methoxyl (HMP) pectins to encapsulate bioactive substances for food and medicinal applications [18].

The final product’s qualities, such as the retention of the active substance, stability, solubility, and antioxidative power, are greatly influenced by the drying technique and wall material chosen. The freeze-drying procedure, in which water from the substance is removed by sublimation, is one often used technique for encapsulating heat-sensitive and unstable chemicals. The procedure used in this method preserves the active chemicals’ original functional characteristics. Additionally, due to the utilization of low temperatures, freeze-drying exhibits positive characteristics in process yield, high-quality products, and anthocyanin preservation [19].

To our knowledge, no investigations on the microencapsulation of yellow onion extracts employing sodium caseinate (SC), pectin (P), and whey protein isolate (WPI) as wall components in the freeze-drying method have been published. The goal of this work was to compare the physicochemical qualities, antifungal capabilities, and shape of several wall material formulations using SC-P-WPI combinations in order to optimize the process of encapsulating yellow onion rejects.

## 2. Materials and Methods

### 2.1. Chemicals

Sulfuric acid, 2,2-Diphenyl-1-picrylhydrazyl (DPPH), sodium thiosulphate, Folin–Ciocalteu reagent (2N), aluminum chloride, sodium hydroxide, sodium acetate, gallic acid potassium chloride, aluminum chloride, sodium hydroxide, ferric chloride heptahydrate, potassium carbonate, sodium acetate, methanol, pectin, whey protein isolates, and sodium caseinate were obtained from Sigma Aldrich (St. Luis, MO, USA).

### 2.2. Onion Preparation

Fresh yellow onion (*Allium cepa* L.) rejects (which were second quality onions, free from serious damages and infections by tops and the minimum size between 5/16 and 1–1/8 inches in diameter) were obtained from a local market in the Province of Khorasan Razavi, Iran. The bulbs were transferred to the laboratory and peeled immediately using a sharp cutter so as to remove the outer dry and semi-dry layers, as well as the apical trimmings. They were dried in hot air at 60 °C for 8 h to a moisture content of under 10% and then pulverized in a domestic blender (approximate mean particle diameter 1 mm). This powder was used for all extraction processes.

### 2.3. Extraction of Compounds from Yellow Onion Rejects Assisted by Pulsed Electric Fields

To extract components from onion rejects, pulsed electric field (PEF) treatment was employed, according to Mahalleh et al. 2019 [20] method with some modifications. A batch-type Pure Pulse PEF system (Technology Food Science, Berlin, Germany) with two parallel stainless electrodes was used for the PEF treatment. Onion reject samples (~10 g) were placed inside a Plexiglas treatment chamber with dimensions of 4 × 10 × 10 cm^3^ between two stainless steel electrodes with 4 cm of gap and area of 10 × 10 cm^2^ and 100 mL of ethanol solvent (1:10 *w*/*v*) was added. The PEF generator (1750 V-20 A, Food Technology, Tehran, Iran) could create an electrical current of 1750 V/cm with exponential decay wave pulses. Pulse generator was set at a voltage of 60 kV/cm and pulse frequency of 1 Hz, and 60 pulses were applied at ambient temperature. In our preliminary studies, different extraction conditions were tested (voltage, 40–60 kV/cm and number of pulses, 20–60), and the desirable treatment condition for the extraction of antioxidant compounds was found to be 60 kV/cm and 50 pulses. Then, the mixture was agitated at 200 rpm for 48 h in dark at ambient temperature. The solvent was removed by vacuum evaporation at 40 °C for 2 h. The crude PFE ethanolic extract (PEFx) of onion was stored at −18 °C until use [21]. Afterward, the extract was then dried in a freeze-dryer at −55 °C at a pressure of 0.15 mmHg for 20 h. The dried samples were stored in the dark at −18 °C until further testing.

### 2.4. Preparation of Microencapsulated Powders

Different amounts of sodium caseinate (SC), pectin (P), and whey protein isolate (WPI) were prepared in distilled water at a concentration of 10 g/100 g of the solution. Compositions of SC, P and WPI mixtures at different design points are listed in Table 1. The solutions were then mixed with a magnetic stirrer (IKA^®^ C-MAG HS 7) (Staufen, Germany) at 3500 rpm for 30 min and refrigerated for 24 h to complete the hydration process [12]. Then the concentrated onion extract was added to the feed at a proportion of 1:5 (onion extract: carrier, *w*/*w*) and homogenized by a rotor-stator homogenizer (IKA T25-Digital Ultra Turrax, Staufen, Germany) at 13,600 rpm for 2 min. Solutions containing coating and core materials (about 200 mL of each sample) were dried in a freeze-dryer (Operon FDB-5503, Gimpo-si, Republic of Korea) at −55 °C at a pressure of 0.15 mmHg for 20 h. Dried samples were stored in the dark at −18 °C until further testing [22].

#### 2.4.1. Total Phenolic Content (TPC)

The total phenolic content of onion extract and microcapsules was determined using the Folin–Ciocalteu method described by Singleton and Rossi (1965) [23]. with slight modifications. For determining TPC of microcapsules, 200 mg of powders were treated with 2 mL of a mixture of ethanol and methanol (1:1). These dispersions were agitated in a Vortex at room temperature for 1 min and then filtered (0.45 mL Millipore Filter, Sigma-Aldrich, St. Luis, MO, USA). Then, 6 mL of double-distilled water and 500 μL of Folin–Ciocalteu reagent was added to 100 μL of the sample extract (with 1:10 volumetric/diluted volumetric methanol), then, after 8 s to 8 min, 1.5 mL of sodium carbonate (20% weight/volume) was added at room temperature. The extract was mixed and allowed to remain for 30 min at 40 °C, and then absorbance was measured at 765 nm using an UV–Vis spectrophotometer (Beckman Instrument DU 600, Fullerton, CA, USA). A mixture of water and reagents was used as blank. A calibration curve of gallic acid (concentration range of 0.04–0.40 mg/mL) in methanol was prepared so that the TPC value could be obtained from the absorbance.

#### 2.4.2. Determination of Total Flavonoid Content (TFC)

The TFC of each sample (filtered extract liquid product) was measured according to the aluminum chloride colorimetric assay [24]. Briefly, 1 mL aliquots of samples or quercetin standards were mixed with 4 mL of Milli-Q water and 0.3 mL of aqueous 5% NaNO_2_. Then 0.3 mL 10% AlCl_3_ and 2 mL 1 M NaOH solution were added after 5 and 6 min, respectively. The total volume was made up to 10 mL with Milli-Q water. The solutions were mixed well, and 200 μL of solution was transferred to 96-well plate in triplicate. The absorbance was measured at 510 nm against a prepared reagent blank using the same microplate reader as above. Total flavonoid content in each extract was determined using a standard curve prepared for quercetin, and the results were expressed as mg quercetin equivalents per gram of dry onion skin (mg quercetin equivalent/g DW).

#### 2.4.3. Radical Scavenging Activity

Free radical-scavenging activity was measured by 2,2-diphenyl-1-picrylhydrazyl (DPPH) assay as described by (Siger et al., 2008) [25]. Discoloration of DPPH was measured at 517 nm with a UV-1601 spectrophotometer (Shimadzu, Kyoto, Japan) using Equation (1):

(1) $$\%\;\mathrm{DPPH}\;=\frac{A_{\mathrm{DPPH}}-A_{S}}{A_{\mathrm{DPPH}}}\;\text{×}\;100$$

where A_S_ is DPPH solution absorbance when the extract has been added at a specified amount and A_DPPH_ is DPPH solution absorbance.

#### 2.4.4. Determination of Encapsulation Yield and Efficiency

The encapsulation yield (Y) of samples after freeze-drying was calculated according to the following formula (Equation (2)) based on dry matter measurements [26].

(2) $$Y=\frac{\;\mathrm{Microencap sulated}\;\mathrm{Powder}\;(g)}{\mathrm{core}\;(g)+\;\mathrm{Wall}\;\mathrm{Materials}\;(g)}\;\text{×}\;100$$

The encapsulation efficiency (EE) was obtained on the method described by (Idham et al., 2012), as shown in Equation (3):

(3) $$\mathrm{EE}=\frac{\mathrm{TPC}-\mathrm{SPC}}{\mathrm{TPC}}\;\text{×}\;100$$

In which TPC is total phenolic content and SPC is surface phenolic content of the freeze-dried microcapsules.

The capsule surface phenols (SPC) were quickly washed with 10 mL of ethanol with a stirrer for 10 s and then centrifuged at 3000 rpm for 3 min at 20 rpm. Then the clear phases were separated by the 0.45-μm microfilter and screened [27]. The amounts of phenolic surface compounds have been calculated and quantified using the same method as defined in the section on total phenolic material.

#### 2.4.5. Bulk Density of the Microcapsules

20 g of microencapsulated powders were transferred to a 20 mL graduated cylinder to determine the bulk density. Bulk density was calculated as the ratio of the powder mass to the volume of the graduated cylinder (kg/m^3^) [28].

#### 2.4.6. Determination of Glass Transition Temperature

For 1 week, samples of freeze-dried powders were equilibrated in a relative humidity of 75%. Glass transition temperature (Tg) was determined using differential scanning calorimetry (DSC) (DSC1 Mettler Toledo, Greifensee, Switzerland). The instrument was calibrated according to the manufacturer’s recommendation. Hermetically sealed aluminum pans were used to avoid any moisture loss during the test. In the experiment, weighed samples (5 mg) were sealed, cooled to −40 °C, held for 5 min, and then heated at a heating rate of 10 °C/min to 200 °C, equilibrated at 200 °C for 5 min, and finally cooled to −40 °C at 10 °C/min [29]. An empty aluminum pan was used as the reference. Re-scans were performed immediately to confirm the existence of a Tg. Then, a temperature scan was performed to obtain Tg values.

#### 2.4.7. Determination of Particle Size Distribution

A laser diffraction particle size analyzer (SALD-2101, Shimadzu, Kyoto, Japan) was used to measure particle size in terms of diameter. The microencapsulated powders were dispersed in hexane using ultrasonic waves (24 kHz and 20% intensity) for 2 min, and then particle size was directly determined [30].

#### 2.4.8. Scanning Electron Microscopy

A scanning electron microscope (LEO 1450; VP, Jena, Germany) was used to observe the particle structures of the prepared microcapsules. For this reason, the sample was placed on the aluminum specimen and the double-sided tape holder. The specimens were then lined with 200 A gold palladium and scanned with electron microscopy at an accelerating voltage of 10 kV [31].

### 2.5. Antifungal Activity

The antifungal activity was tested by disc diffusion method (Taylor et al., 1995). Potato dextrose agar plates were inoculated with fungal culture (10 days old) by filter paper discs (7 mm diameter) impregnated with the concentration of sodium nitrate (1000 mg/178 L) and the extract (1000–3000 mg/L) and placed on test organism-seeded agar plates. Ethanol was used to dissolve the extract (onion extract at optimum extraction condition) and was completely evaporated before application on test organism-seeded plates. Blank disc impregnated with solvent Ethanol followed by drying off was used as negative. The activity was determined after 72 h incubation at 25 °C. The diameters of the inhibition zones were measured in mm [32].

### 2.6. Experimental Design and Statistical Analysis

In the current study, the simplex (Type of lattice) lattice mixture design according to the procedures described by Mahalleh et al. (2021) [29].was applied to evaluate the effect of sodium caseinate (SC), pectin (P), and whey protein isolate (WPI) on the physico-chemical characteristics of the dried and microencapsulated powder. The dependent variables (responses) and physicochemical properties of encapsulated powders were analyzed, and the fitted models were subjected to variance analysis (ANOVA) to specify significance (*p* < 0.05), determination coefficient (R^2^), and lack of fit. Lack of fit value confirms the applicability of the model. The best-fit equations for all the responses were established after removing the non-significant terms. Multiple response optimizations were conducted to recognize the combination of experimental factors, which at the same time optimize the responses. Component proportions were expressed as fractions of the mixture with a sum (A + B + C) of 100. These three factors: Sodium Caseinate, Pectin, and Whey protein isolate, levels, and experimental design in terms of coded and uncoded as 14 combinations are presented in Table 1. Design Expert 10.0 software (Statease Inc., Minneapolis, MN, USA) was used for regression analysis and to generate 3D surface plots. The probability levels of *p* ≤ 0.01 and *p* ≤ 0.05 were considered to be significant for statistical procedures. The desirability method detailed in Derringer and Suich (1980) [33]. was applied to find the best combination of wall materials that will result in rejected onion extract with optimum values for the dependent variables (EY, TPC, TFC, and DPPH). Desirability ranges from zero to one for any given response. A value of one represents the ideal case, while zero indicates that one or more responses fall outside the desirable limits.

Numerical optimization was used to find the best conditions for onion-rejected extract microencapsulation on Responses. Statistical analysis experiments aim to maximize encapsulation yield, total phenolic content, and total flavonoid content as well as free radical scavenging capacity. Some graphs were drawn in Excel (Microsoft Office 2007), and some by Design Expert 11.0. All measurements and trials were performed in triplicates.

## 3. Results and Discussion

### 3.1. Physicochemical Properties of Encapsulated Powders

**Figure 1:**
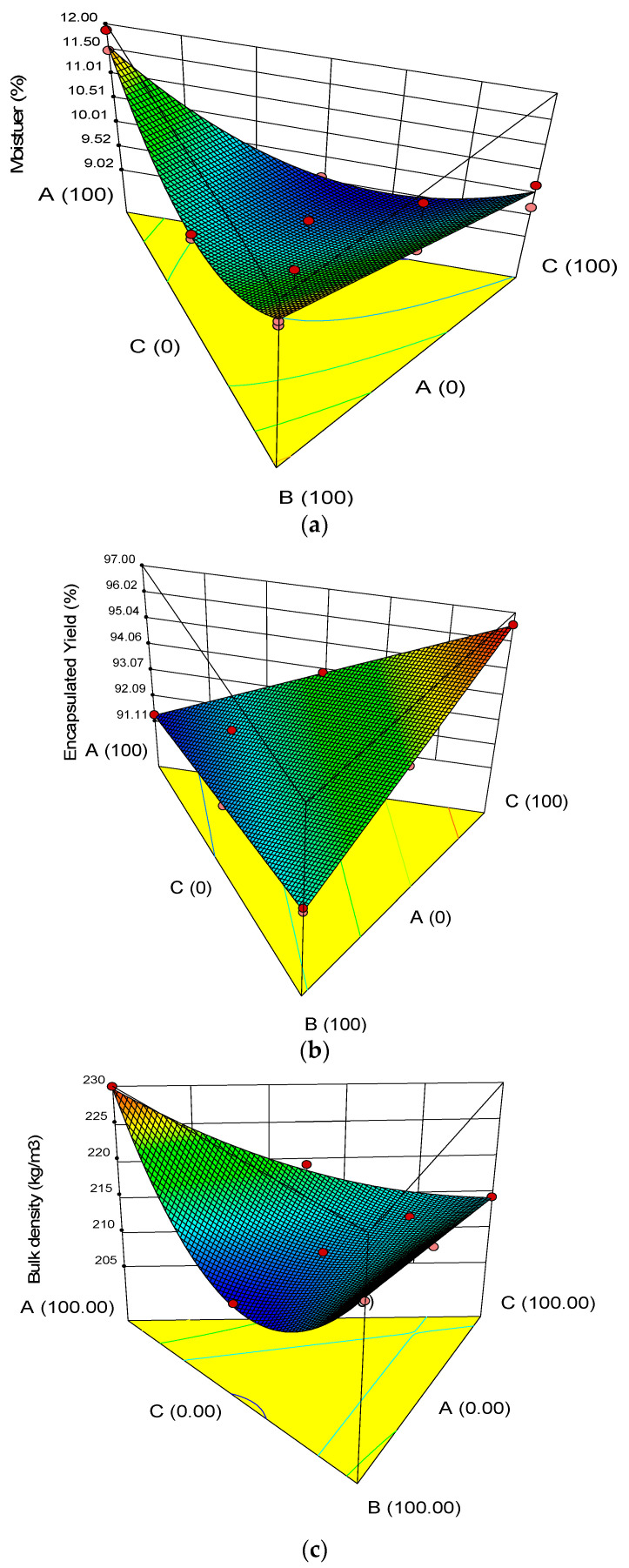
Estimated response surface plots indicating effect of sodium caseinate (SC), pectin (P), and whey protein isolate (WPI) levels on physical parameters: (**a**) Moisture, (**b**) Encapsulation Yield, (**c**) Bulk Density.

Mean values for the responses (moisture, encapsulation yield, bulk density, particle size, glass transition temperature) were calculated, and the corresponding results are shown in Table 2. The experimental results obtained for all studied parameters were statistically analyzed by fitting the data to the various models, and the evaluated regression models were recorded (Table 3). The model with a maximum R^2^ and *p* value less than 0.05 were selected. Three-dimensional surface plots indicating the influence of the wall materials on the exclusive responses are illustrated in Figure 1a–c. All models were statistically significant (*p* < 0.05), showing a good relationship between the responses and the ingredients (factors) at the 95.0% confidence level (Table 3). R^2^ values of the established models were higher than 0.93, illustrating that they were relatively adequate for the prediction aim.

#### 3.1.1. Moisture Content

The moisture content of the microencapsulated onion extract ranged between 9.59 and 11.88%, which were within the acceptable range of microencapsulated powder (0.9–12%) [29]. (Table 2). Higher concentrations of CS (100%) considerably increased the moisture content (Table 2 and Figure 1a). The regression equation for moisture content response in terms of L Pseudo components values, obtained from mixture design, is stated in Equation (4).

Y = 11.58SC + 11.59P + 10.02WPI − 5.22SC × P − 4.79ACSC × WPI(4)

During the encapsulation of bioactive compounds, the combined influence of various types of wall materials on encapsulation needs to be considered. There could be differences in the moisture content of the final product, even though they have the same initial moisture. This variation in moisture contents could be attributed to the chemical structure and affinity of wall materials to water (SC, P, and WPI). The hydrocolloid materials used have hydrophilic groups which can easily bind different fractions of water molecules and result in higher moisture retention [34]. In addition, the water-holding properties of the structure and porosity of the powder particles during drying can be the other considerations for the moisture contents of the powder. Stoica et al. (2022) [8] observed a similar behavior, studying the freeze-drying of red onion skin with different combinations of wall materials.

Because a large level of moisture induces particle cohesion and accelerates microbiological development and oxidation, moisture plays an important role in the powder’s stability [22]. Moisture is a significant quality that influences drying efficiency, powder fluidity, stickiness, and storage stability [35]. Higher CS concentrations (100%) resulted in a significant rise in moisture content (Table 2 and Figure 1a). Aidoo et al. (2014) [36] found that high quantities of CS resulted in greater moisture content. The microcapsules with 100 percent CS and P had the most moisture (*p* < 0.05), which is likely due to the difference in the number of hydrophilic groups in the molecules of the wall compositions [37]. 

#### 3.1.2. Encapsulation Yield

According to Table 2, microcapsule efficiency varied from 78.4 to 98.4%. Results from the analysis of variance signaled that the type of wall composition had a statistically significant effect on the micro capsulation efficiency (*p* < 0.05). Comparing the treatment means showed that the micro capsulation efficiency in capsules prepared with a mixture of sodium caseinate (50%), pectin (50%) coatings were higher. The microencapsulation yield of onion extract was between 84.7 and 99.7% (Table 2). Overall, increasing CS and WPI levels ended in an increase in the EY (Figure 1b and Table 2). The relationship between the wall materials (SC, P, and WPI) and EY was quadratic, and the highest EY was obtained in chocolate samples containing 100% SC and P. The regression equation for EY response in terms of L-pseudo components values, obtained from mixture design, is presented in Equation (5). The equation in terms of coded factors can be used to make predictions about the response for given levels of each factor. By default, the high levels of the mixture components are coded as +1, and the low levels are coded as 0. The coded equation is useful for identifying the relative impact of the factors by comparing the factor coefficients.

Y = 91.35SC + 92.85P + 96.56WPI(5)

The efficiency in the plot (Figure 1a–c) showed that increasing the amount of pectin from the center point of the triangle design (where the three points have equal ratios) up to 33.3% increased the efficiency during storage and then decreased. According to the graph, the reduction of sodium caseinate or whey protein isolate isolated in the combination boosted microcapsule production efficiency. When protein and carbohydrates are combined in a microcapsule, the core material is better protected, and encapsulation efficiency is improved. As a result, the combination of the amphiphilic protein molecule with pectin provides greater microencapsulation and stability. The greater the ratio of pectin to whey protein isolate, the greater the synergistic impact, resulting in increased encapsulation efficiency [38].

#### 3.1.3. Bulk Density

A general trend emerged that an increase in WPI level led to an increase in bulk density value (Figure 1c and Table 2). The term associated with the interaction between the effects of three components was significant (*p* < 0.01) (Table 3). The bulk density ranged from 208 to 232 percent. The difference in bulk density between samples was found to be significant (*p* < 0.05). The microcapsules made with P had the greatest bulk density (100). The bulk density of powders is affected by the size, fragility, and fluidity of crushed particles. The molecular weight of the wall materials determines the bulk density of the powders, and less overall volume raises the bulk density [7]. Equation (6) represents the regression equation for bulk density response in terms of L Pseudo components values generated through mixture design.

Y = 229.67SC + 222.23P + 214.50WPI − 64.44SC × P − 14.08SC × WPI(6)

#### 3.1.4. Particle Size, Glass Transition, and the Scanning Electron Microscopy

The average particle size (μm) of microcapsules obtained with various wall materials is shown in Table 2. The particle size of microcapsules ranged from 31.2 to 43.5 μm. The size of the microscopic particles in the capsule is mostly determined by the type of material used in the capsule’s wall. The size of freeze-dried microcapsules has been observed to range from 20 to 5000 μm. The sublimation of ice crystals and longitudinal fractures in this process cause variable particle sizes [39]. Condurache et al. (2019) reported that the microencapsulated powders’ size ranges were from 8.5 to 81.2 μm.

One of the best methods for testing the thermal behavior of materials is the use of differential scanning calorimetry (DSC). The glass transition temperature can also be calculated by DSC. In this method, the specimen is subjected to a controlled temperature change, and the changes in its physical properties are continuously measured as a function of temperature [40]. At temperatures below the glass transition point, matter molecules are not motile, resulting in a glassy and hard substance. Above the glass transition temperature, the polymer has a rubber and soft mode (the beginning of molecular movements in the polymer). The core materials can release and transfer out of the wall materials when the polymer is in the rubber and soft mode (temperatures above the glass transition temperature).

**Figure 2:**
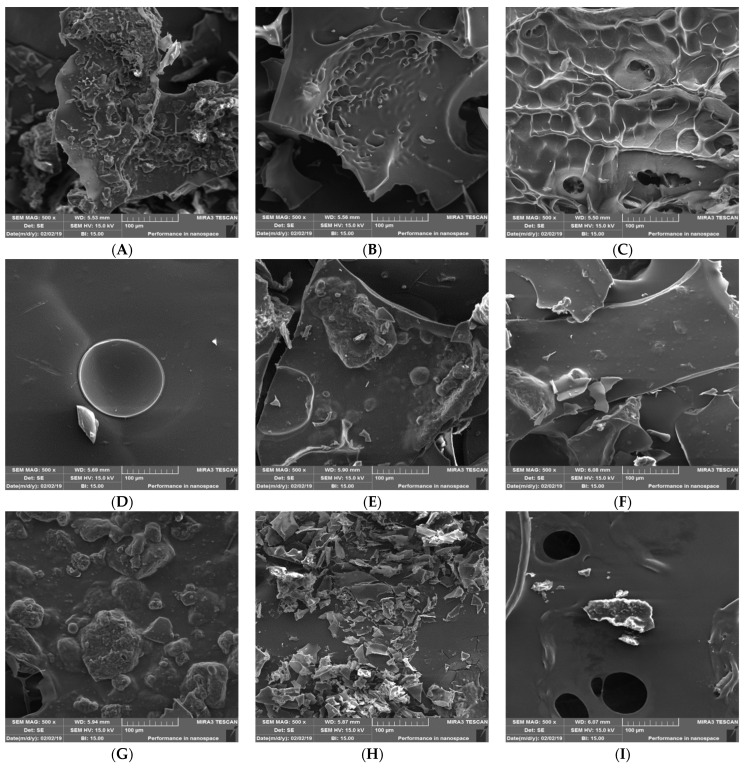

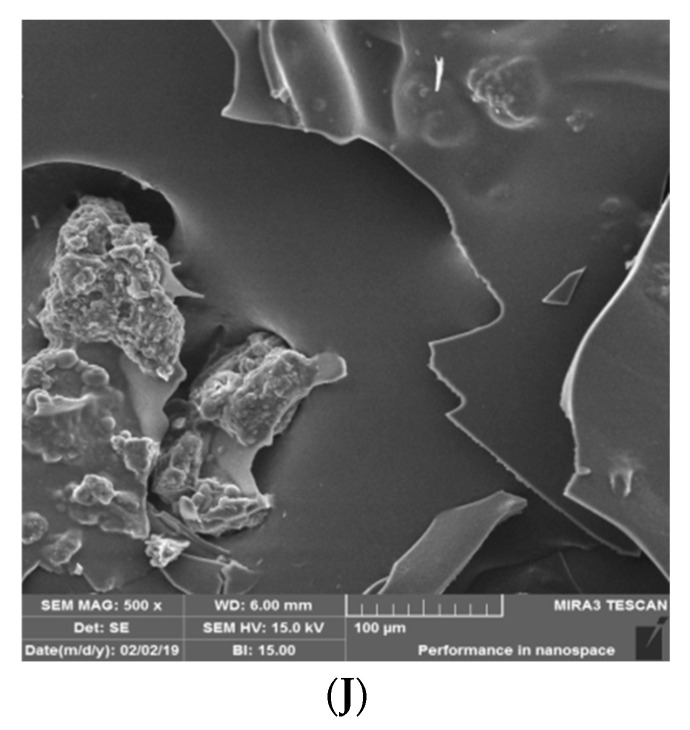
Scanning electron micrographs of freeze-dried encapsulated powders. (**A**): CS (50), P (50), WPI (0); (**B**): CS (0), P (50), WPI (50); (**C**): CS (0), P (0), WPI (100); (**D**): CS (50), P (0), WPI (50)); (**E**) CS (100), P (0), WPI (0); (**F)**: CS (16.7), P (66.7), WPI (16.7); (**G**): CS (0), P (100), WPI (0); (**H**): CS (16.7), P (16.7), WPI (66.7); (**I**): CS (66.7), P (16.7), WPI (16.6); (**J**): CS (33.3), P (33.3), WPI (33.3).

As can be seen in scanning electron microscope images (Figure 2), there was no definite geometric form for different types of prepared microcapsules. This could be due to the combination of pressure and temperature during the freeze-drying process. Ice crystals can be sublimated immediately without changing phase, resulting in solids that are sponge-like, brittle, and flaky. Solids are detected in coated and laminated forms when ice is sublimated. Furthermore, Fernandes et al. (2012) [6] stated that the smooth, with dents and slight agglomeration, surfaces of the powders are characteristic of encapsulated phytochemicals and indicate the encapsulating agent’s suitability.

As can be seen in Figure 2, the wall materials had a smooth but non-uniform coating. The smallest amount of agglomeration and curvature on the surface, less porous structure, larger size, and better distribution confirmed this compound as the compound of encapsulation.

The formation of surface wrinkles and cavities on the surface of the microcapsule probably indicates the effect of mechanical stress and drying conditions on the wall materials. The composition of the wall and the drying rate, especially in the early stages, can affect the microstructure surface properties of the encapsulated matter. Mahalleh et al. (2021) [29] reported that Arabic gum microcapsules and maltodextrin in wall materials showed brittle and dentate forms on the surface. The shrinkage and agglomeration mode showed poor encapsulation properties of these wall materials for phenols.

### 3.2. Chemical Properties of the Microcapsules

Mean values for the responses (Encapsulation Efficiency, TPC, TFC, and DPPH) were calculated, and the corresponding results are shown in Table 4. The experimental results obtained for all studied parameters were statistically analyzed by fitting the data to the various models, and the evaluated regression models were recorded (Table 5). The model with maximum R^2^ with *p* value less than 0.05 was selected. Three-dimensional surface plots indicating the influence of the wall materials on the exclusive responses are illustrated in Figure 2A–D. All models were statistically significant (*p* < 0.05), showing a remarkable relationship between the responses and the ingredients (factors) at the 95.0% confidence level (Table 5).

The encapsulation efficiency is defined as the percentage of the active ingredient that is successfully captured in the carrier material of the microcapsule. It ranged from 77.1 to 86.7% in the prepared microcapsules. The encapsulating wall materials used had a significant impact on the EE of the samples. As can be seen in Figure 2A, EE increased by increasing SC and WPI, while there was a slight decrease by increasing P. The combination of 50% SC and 50% WPI had the lowest encapsulation efficiency. The features of the coating and the core material, such as emulsifying capabilities and drying parameters, are among the elements that influence the microencapsulation efficiency of phenolic compounds [41]. Some other studies recorded 59.1–71.3% encapsulation efficiency values for the encapsulation of flavonoids from yellow onion skins in whey protein as a wall material [42], 69.9–77.6% for anthocyanins in a mixture of pectin, carboxymethylcellulose and whey proteins as a wall [43]. Such findings suggest that in microencapsulation, a single form of wall material cannot provide all the required properties, and a mixture of carbohydrates in the wall with proteins and polysaccharides contributes to optimum efficiency. The selection of coating material is determined by the ratio of the core material, which acts as an important factor in producing uniform microcapsules with high encapsulation efficiency. Thus, the choice of wall material is fundamental and should be biocompatible and have food-grade status [44]. During the encapsulation of bioactive compounds, the combinations of various types of wall materials might be considered. According to Klein et al. (2010) [45], interactions between diverse wall materials might result in complexes with interfacial and amphiphilic features, which improve encapsulation efficiency.

The phenolic compound amount ranged from 45.23 to 54.24 mg/g (Table 4). The results showed that the encapsulation efficiency of phenolic compounds improved significantly in the combination of WPI and P inside the wall. As regards the form of wall content, the highest rates of phenolic compounds were in microcapsules with wall formula P and WPI (50 and 50). This indicates that the combination of WPI and P is suited to protect phenolic compounds. The TFC amount ranged from 76 to 94.65 mg/g (Table 4). As regards the form of wall content, the highest rates of TFC were in microcapsules with wall formula CS and WPI (50 and 50). In a previous study, Milea et al. (2020) [46] encapsulated flavonoids from yellow onion skins using maltodextrin, pectin, and whey protein hydrolysates as coating materials in different ratios. The concentration of flavonoids in the freeze-dried variants varied from 98.1 ± 0.5 to 103.7 ± 0.6 mg QE/g DW.

Horincar et al. (2019) [42] used different combinations of biopolymeric coatings based on whey protein isolate and chitosan, maltodextrin, and pectin as adjuvants for encapsulation. These authors obtained two variants of freeze-dried powder with different profiles. Therefore, lower values for the total flavonoid content of 5.8 ± 0.2 mg QE/g DW and antioxidant activity of 175.9 ± 1.5 mM TE/g DW were suggested in coatings with WPI-chitosan. When using a more complex biopolymeric wall material, including WPI-maltodextrin-pectin, these authors obtained a powder with significantly higher flavonoid content and antioxidant activity of 104.9 ± 5.0 mg QE/g DW and 269.2 ± 3.6 mM TE/g DW, respectively

**Figure 3:**
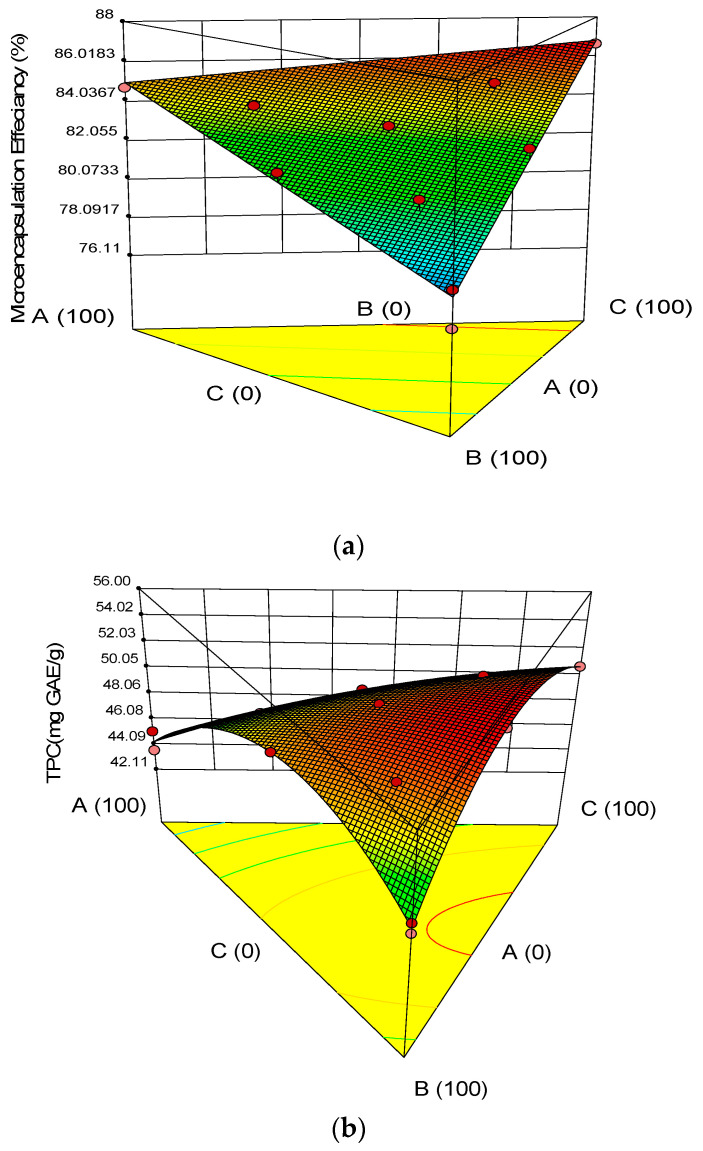

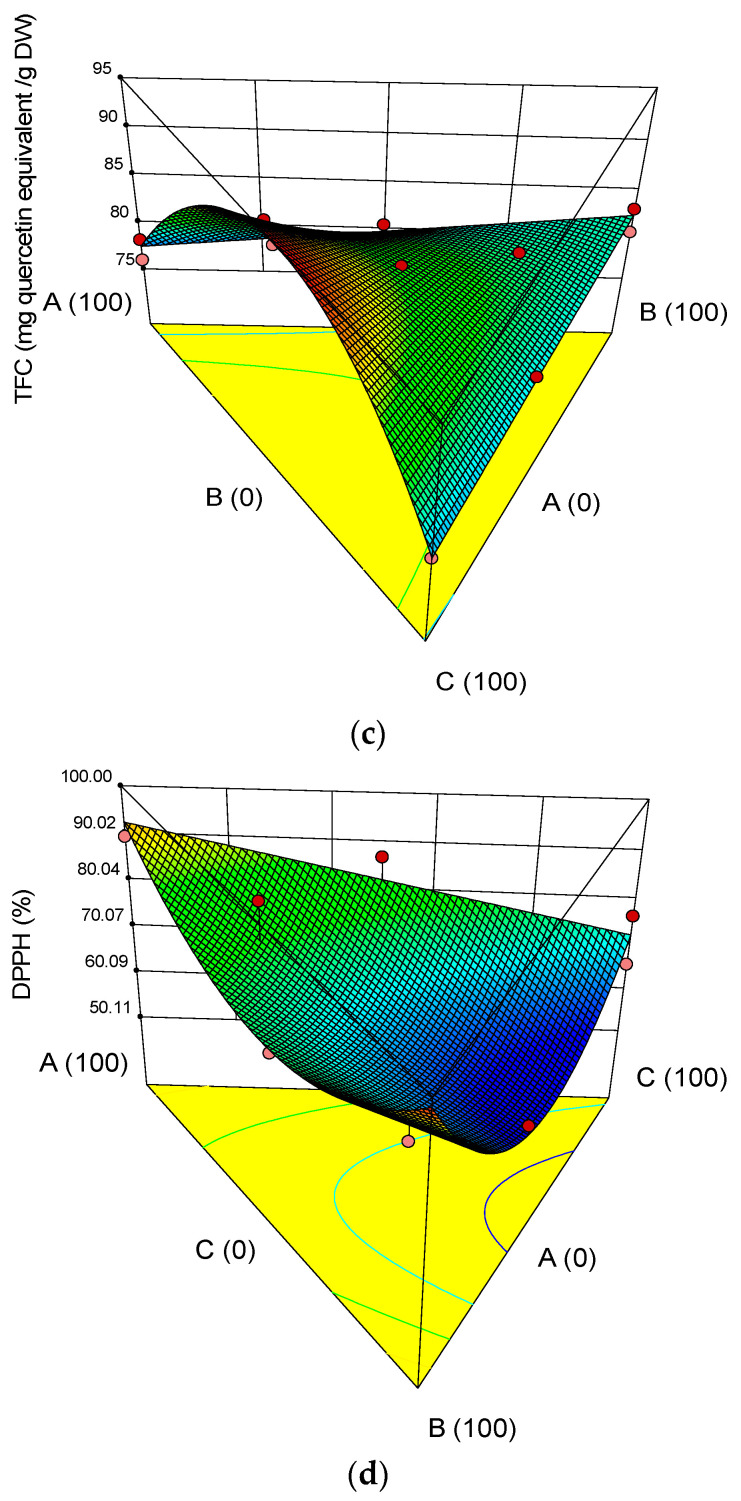
Estimated response surface plots indicating effect of sodium caseinate (SC), pectin (P), and whey protein isolate (WPI) levels on physical parameters: (**a**) Microencapsulation Efficiency, (**b**) TPC, (**c**) TFC, (**d**) DPPH, Free radical scavenging capacity in encapsulated freeze-dried onion’s extract.

According to Table 4, the amount of DPPH of microcapsules varied from 63.64 to 99.33%. Analysis of variance results showed that the type of coat in microencapsulation had a significant effect on antioxidative properties. The DPPH was the highest in microcapsules with a 100% pectin wall. As can be seen in Figure 3d, the free radical scavenging capability increased by increasing the amount of SC and decreased by increasing P and WPI up to the center point, and the DPPH increased by increasing P and WPI. Bourvellec and Renard (2012) [47] also explained that this phenomenon appears due to the ability of polysaccharides to encapsulate phenolic compounds, competing with the binding to proteins. They also stated that ionic polysaccharides such as pectin are known as effective inhibitors of polyphenol/protein complexation. The types of wall and core compounds have been determined to have a major influence on the protective effect of microencapsulated compounds [48]. Moreover, the molecular size of the wall used plays an important role in removing the nucleus compounds as they directly influence the molecular distribution of the compounds around the microcapsules into the shell and their removal to the surface [49].

### 3.3. Optimization and Validation

The desirability function method described in the Section 2 was employed to analyze the process parameters concerning the dependent variables. The desired levels for each of the operational conditions (SC and WPI) were selected within the range, and P target 10% while the dependent variables (EY, TPC, TFC, DPPH) were defined as maximum. Each of the dependent variables was analyzed separately. The optimal conditions were obtained by Design-Expert software.

**Figure 4:**
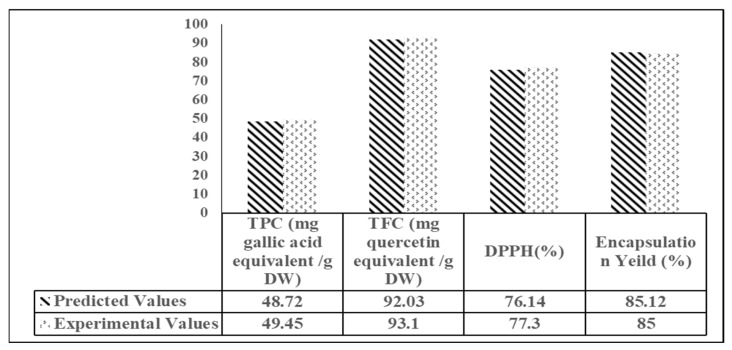
Predicted and experimental results used in the validation of model based on optimum formulations Sodium caseinate (SC) = 47.58%, Pectin (P) = 10.00%, and whey protein isolate (WPI) = 42.42%.

The final result of this optimization suggested that the best wall materials were 47.6 g/100 g (SC), 10.0 g/100 g (P), and 42.4 g/100 g (WPI), with encapsulation yield (EY) of 85.1%, with total phenolic content (TPC) of 48.7 mg gallic acid equivalent/g DW, total flavonoid content (TFC) of 92.0 mg quercetin equivalent/g DW, and DPPH capacity of 76.1%, respectively. To check that the conditions were accurate, the experiment was repeated under ideal conditions, and the results are reported in Figure 4. The models’ efficiency was demonstrated by the lack of significant deviations between the models and the experimental observations (*p* < 0.05).

### 3.4. Antifungal Activity

The effect of various amounts (5 to 25 L) of onion extract (concentration 3000 mg/L) was obtained under optimal processing conditions (wall materials were 47.6 g/100 g (SC), 10.0 g/100 g (P), and 42.4 g/100 g (WPI)) on the inhibition of *A. Niger* after 72 h of storage at 25 °C, as well as the diameter of the inhibition zone in comparison to Sodium Nitrate (120 ppm) as a synthetic preservative, are shown in Table 6. The mean diameter of the inhibitory zone against *A. Niger* increased when the concentration of onion extract was raised from 1000 to 3000 mg/L. Furthermore, the antifungal action of onion extract at 3000 mg/L (18.63 mm) did not differ substantially from that of sodium nitrate (18.8 mm) (*p* < 0.05). For a wide spectrum of molds and yeasts, sodium nitrate is a good preservative. Suppression of dehydrogenase enzymes in fatty acid oxidation, catalase inhibition, and potential uncoupling of oxidative phosphorylation by inhibition of enzymes, especially sulfhydryl-containing enzymes, are thought to be the causes of this preservation activity [50]. The phenolic chemicals in onions are likely to be the cause of their extraction inhibitory activity. The effect of phenolic compounds in suppressing the growth of bacteria and fungi varies depending on the phenolic compounds and their quantity, as well as the extraction process (the solvent used for the extraction). Ibrahium (2010) [51] reported that the methanolic extract of onion inhibits the growth of *Aspergillus Niger.*

## 4. Conclusions

In this work, the bioactive components from rejected onions were extracted using the pulse electric field process extraction method. With a mass ratio of 1:5 (extract/wall material, *w*/*w*), the onion extract was encapsulated with whey protein isolate, pectin, and sodium caseinate. A simplex lattice design with enhanced axial points in mixture design was used to optimize the wall materials. The model was significant at the ideal conditions, and the highest encapsulation yield, total phenolic content, total flavonoid content, and DPPH capacity were obtained. The optimal wall materials (47.6 g/100 g (SC), 10.0 g/100 g (P), and 42.4 g/100 g (WPI)) had an antifungal effect on *Aspergillus niger*. These findings are expected to be helpful for further exploitation and application of sustainable resources.

## Figures and Tables

**Table 1:** Experimental design and mass fraction of three wall components according to simplex lattice mixture design.

Mixtures	Uncoded Values (Ingredient Proportions)		
	CS (%)	P (%)	WPI (%)
1	16.67	16.67	66.67
2	0	0	100
3	0	100	0
4	0	100	0
5	50	50	0
6	0	50	50
7	0	0	100
8	50	0	50
9	100	0	0
10	66.67	16.67	16.67
11	100	0	0
12	50	50	0
13	33.33	33.33	33.33
14	16.67	66.67	16.67

SC: sodium caseinate, P: pectin, WPI: whey protein isolate.

**Table 2:** Different wall material treatments based on mixture design to encapsulate onion extract and physical properties of encapsulated powders.

Mixtures	Physical Properties of Encapsulated Powders				
	M (%)	EY (%)	BD (kg/m^3^)	PS (μm)	Tg (°C)
1	10.2 ± 0.31	96.5 ± 0.76	215.1 ± 2.35	33.2 ± 0.35	28.1 ± 0.11
2	9.7 ± 0.24	96.1 ± 0.46	214.5 ± 3.58	-	-
3	11.5 ± 0.15	96.8 ± 0.67	221.7 ± 6.57	-	-
4	11.5 ± 0.38	98.2 ± 0.49	223.0 ± 5.92	31.2 ± 0.54	41.1 ± 0.02
5	10.2 ± 0.12	84.7 ± 0.42	210.3 ± 4.42	-	-
6	10.8 ± 0.42	99.7 ± 1.01	217.8 ± 8.56	43.5 ± 0.67	27.1 ± 0.32
7	10.2 ± 0.54	95.0 ± 1.42	214.5 ± 5.47	-	-
8	9.6 ± 0.64	96.8 ± 0.96	219.3 ± 6.54	-	-
9	11.5 ± 0.33	97.2 ± 0.95	229.9 ± 0.92	-	-
10	9.6 ± 0.52	91.5 ± 1.09	215.0 ± 9.36	35.4 ± 0.66	27.2 ± 0.12
11	11.9 ± 0.16	93.9 ± 0.82	229.9 ± 9.65	36.4 ± 0.47	41.2 ± 0.52
12	10.3 ± 0.22	86.0 ± 0.69	210.3 ± 10.48	34.6 ± 0.65	28.8 ± 0.65
13	10.2 ± 0.14	88.0 ± 0.88	213.7 ± 8.46	-	-
14	10.8 ± 0.12	88.4 ± 0.83	214.5 ± 10.66	-	-

M: Moisture; EY: Encapsulation yield; BD: Bulk density; PS: Particle size; Tg: Glass transition temperature. Mean ± SD (standard deviation) within a column.

**Table 3:** Regression models for physical parameters of onion extract.

Fitted Model	Moisture (M)	Encapsulated Yield (EY)	Bulk Density (BD)
	Quadratic (*p* ≤ 0.001)	Linear (*p* ≤ 0.0001)	Quadratic (*p* ≤ 0.03)
Lack of fit	*p* = 0.191	*p* = 0.972	*p* = 0.060
R Squared (%)	0.89	0.99	0.98
Adjusted R Squared (%)	0.84	0.99	0.97
Adeq Precision	11.0	282	36.9

**Table 4:** Chemical properties of encapsulated powder.

Mixtures No.	Chemical Properties of Encapsulated Powders			
	Encapsulation Efficiency (%)	TPC (mg Gallic Acid Equivalent/g DW)	TFC (mg Quercetin Equivalent/g DW)	DPPH (%)
**Onion Extract**	**-**	**65.34 ± 4.43**	**98.45 ± 4.438**	**99.89 ± 3.45**
1	85.25 ± 1.23	52.48 ± 0.43	86.87 ± 1.33	63.64 ± 2.44
2	86.65 ± 3.43	50.56 ± 0.68	79.58 ± 0.85	65.37 ± 1.34
3	78.90 ± 0.45	49.06 ± 0.33	82.73 ± 0.96	98.26 ± 2.64
4	77.12 ± 0.75	48.23 ± 0.75	80.23 ± 1.17	99.33 ± 1.89
5	82.23 ± 1.15	52.37 ± 0.69	80.52 ± 2.75	74.79 ± 0.95
6	83.20 ± 0.37	54.24 ± 0.65	81.15 ± 4.35	60.12 ± 0.64
7	86.65 ± 0.67	50.56 ± 0.38	79.58 ± 2.47	75.98 ± 0.95
8	85.62 ± 0.46	48.66 ± 0.53	94.65 ± 1.76	87.00 ± 1.66
9	84.71 ± 0.63	45.23 ± 0.27	78.31 ± 1.66	89.82 ± 0.94
10	84.27 ± 0.45	49.57 ± 0.62	86.24 ± 0.18	86.56 ± 1.66
11	84.71 ± 0.87	43.69 ± 0.87	76.00 ± 0.69	89.82 ± 1.54
12	82.23 ± 0.63	52.37 ± 1.15	80.52 ± 0.85	74.79 ± 0.84
13	83.77 ± 0.37	53.08 ± 2.11	87.18 ± 1.45	66.78 ± 1.38
14	81.53 ± 0.54	53.22 ± 1.18	83.21 ± 1.18	67.40 ± 1.65

TPC: Total Phenolic Content, TFC: Total Flavonoid Content, DPPH: Free radical-scavenging activity was measured by 2,2-diphenyl-1-picrylhydrazyl. Mean ± SD (standard deviation) within a column.

**Table 5:** Regression models for chemical parameters of onion extract.

	Encapsulation Efficiency	TPC	TFC	DPPH
Fitted Model	Linear (*p* ≤ 0.0001)	Quadratic (*p* ≤ 0.0001)	Quadratic (*p* ≤ 0.0001)	Quadratic (*p* ≤ 0.0002)
Lack of fit	*p* = 0.672	*p* = 0.994	*p* = 0.995	*p* = 0.263
R Squared (%)	0.96	0.98	0.917	0.91
Adjusted R Squared (%)	0.95	0. 97	0.96	0.87
Adeq Precision	31.32	33.72	35.15	14.21

**Table 6:** The Effect of onion extract, Sodium Nitrate, and control on the diameter of inhibition zone of *A. niger*.

Samples	The Mean Diameter of Inhibition Zone
Onion Extract (mg/L)	
1000	10.18 ± 0.12c
2000	11.76 ±0.04b
3000	18.63 ± 0.19a
Sodium Nitrate(120 ppm)	18.78 ± 0.04a
Ethanol 70% (negative control)	-

Mean ± SD (standard deviation) within a column sharing the same letter are not significantly different at *p* > 0.05.

## Data Availability

The data presented in this study are available on request from the corresponding author.
